# Identification of novel gout loci from trans-ethnic meta-analysis of serum urate level

**DOI:** 10.1007/s13577-024-01128-0

**Published:** 2024-11-10

**Authors:** Yusuke Kawamura, Akiyoshi Nakayama, Masahiro Nakatochi, Yuka Aoki, Yu Toyoda, Takahiro Nakamura, Seiko Shimizu, Keitaro Matsuo, Nariyoshi Shinomiya, Hirotaka Matsuo, for Japan Gout Genomics Consortium (Japan Gout), Ken Yamamoto, Toru Shimizu, Hiroshi Ooyama, Keiko Ooyama, Mitsuo Nagase, Yuji Hidaka, Tappei Takada, Kimiyoshi Ichida, Kenji Wakai, Takashi Tamura, Miki Ueno, Kimiko Hayano, Yuzo Takada, Hiroshi Nakashima, Mitsunobu Tanaka, Noriyuki Yoshioka, Satoko Iwasawa, Masashi Tsunoda, Kyoko Morichika, Miho Miyazawa, Mayuko Nakajima, Kazuki Maehara, Mana Kirihara, Yuka Aoyagi, Shin Fujiwara, Yurino Mori, Risa Tanaka, Mio Horie, Masumi Someya

**Affiliations:** 1 Department of Integrative Physiology and Bio-Nano Medicine, National Defense Medical College, Tokorozawa, Japan; 2 Public Health Informatics Unit, Department of Integrated Health Sciences, Nagoya University Graduate School of Medicine, Nagoya, Japan; 3 Laboratory for Mathematics, National Defense Medical College, Tokorozawa, Japan; 4 Division of Cancer Epidemiology & Prevention, Aichi Cancer Center, Nagoya, Japan; 5 Division of Cancer Epidemiology, Nagoya University Graduate School of Medicine, Nagoya, Japan; 6 Department of Biomedical Information Management, National Defense Medical College Research Institute, National Defense Medical College, Tokorozawa, Japan

**Keywords:** Pyruvate kinase, Lactate, URAT1/SLC22A12, Gout/hyperuricemia, Urate/uric acid

## To the Editor

Gout is a well-known inflammatory arthritis characterized by sudden, severe pain attacks. It is caused by persistent hyperuricemia and the accumulation of monosodium urate crystals (MSU) in the joint, leading to inflammation and intense pain [1]. To date, we have identified multiple loci associated with serum urate levels [2] and clinically defined gout [1] from genome-wide association studies (GWASs). Recently, after 28 loci were selected from 36 loci identified by the GWAS of serum urate levels in 121,745 Japanese individuals [2], nine loci were reported to be significantly associated with clinically defined gout [3], using the results of the previous gout GWAS [1].

In the present study, to identify further gout loci, we analyzed more selected loci from the results of the trans-ethnic meta-analysis of serum urate levels in 232,092 individuals [2], which discovered 59 statistically significant loci. Of these, 42 urate-related loci were selected for the present association study of clinically defined gout, since the two previous reports [1, 3] identified a total of 17 loci associated with clinically defined gout (*ABCG2*, *CUX2 (ALDH2)*, *SLC2A9*, *SHLD2*/*FAM35A*, *GCKR*, *NRXN2*, *SLC17A1*, *BCAS3*, *UNCX-MICALL2*, *BICC1*, *EMX2-RAB11FIP2*, *NFAT5, PDZK1*, *LRP2*, *PRDM8-FGF5*, *MLXIPL* (*BAZ1B*), *IGF1R*). Supplementary Table S1 shows the results of the association analysis of clinically defined gout for 42 candidate loci. These results were obtained by the summary data of our previous gout GWAS [1], in which 3053 male cases with clinically defined gout and 4554 normouricemic male controls (with no gout history and serum urate ≤ 7.0 mg/dl) were analyzed. The level of significance α was set to a *p* value of < 1.19 × 10^–3^ (= 0.05/42 with Bonferroni correction).

As shown in Table 1, the present study revealed five loci that were significantly associated with clinically defined gout. Of these five loci, two loci (*ORC4* and *MYO9A*) were identified as novel gout loci, and *NRG4* was firstly reported to be associated with clinically defined gout in the present study (Table 1). Two remaining loci, *A1CF* and *MLXIP*, were reported in our other previous papers to be related to clinically defined gout [4, 5]. The results of three loci (*ORC4*, *MYO9A*, *NRG4*) were as follows: rs2307394 [a missense variant, p.Asn78Ser, in *origin recognition complex subunit 4* (*ORC4*)] (*p* value = 2.80 × 10^−5^; odds ratio (OR): 1.15; 95% confidence interval (CI) 1.08—1.24), rs2957742 [an intronic variant in *myosin IXA* (*MYO9A*)] (*p* value = 3.54 × 10^−4^; OR: 1.13; 95% CI 1.06—1.21), and rs4886755 [an intronic variant in *neuregulin 4* (*NRG4*)] (*p* value = 6.83 × 10^−4^; OR: 1.13; 95% CI 1.05—1.21). More detailed information on the above three loci (*ORC4*, *MYO9A*, *NRG4*) are provided in the Supplementary Discussion. Here, we briefly describe the association of gout and each locus.

**Table 1:** Five loci associated with clinically-defined gout identified in the present study

SNP^a^	Locus	Chr	Position^b^	*Gene*	Alleles		Trans-ethnic meta-analysis (SU)^c^		Association analysis (Gout)^d^	
					Risk^e^	Non-risk	Log_10_BF^f^	Posterior probability	OR (95%CI)	*p* value^g^
**rs2307394**	**2q23.1**	**2**	**148,716,428**	***ORC4 (ACVR2A)***	**C**	**T**	**6.72**	**0.480**	**1.15 (1.08, 1.24)**	**2.80 × 10**^**–5**^
rs10994856	10q11.23	10	52,645,248	*A1CF*	A	G	13.23	0.024	1.31 (1.13, 1.52)	2.78 × 10^–4^
rs7953704	12q24.31	12	122,625,992	*MLXIP*	G	A	9.66	0.006	1.14 (1.06, 1.21)	2.08 × 10^–4^
**rs2957742**	**15q23**	**15**	**72,302,894**	***MYO9A (PKM)***	**C**	**G**	**8.54**	**0.027**	**1.13 (1.06, 1.21)**	**3.54 × 10**^**–4**^
**rs4886755**	**15q24.2**	**15**	**76,298,132**	***NRG4***	**G**	**A**	**12.31**	**0.010**	**1.13 (1.05, 1.21)**	**6.83 × 10**^**–4**^

^a^ dbSNP rs number

^b^ SNP positions are based on NCBI human genome reference sequence Build hg19

^c^ Results of trans-ethnic meta-analysis of SU were obtained from Ref.2 (Nakatochi, et al., ***Commun Biol***, 2019)

^d^ Results of genome-wide meta-analysis of Japanese clinically-defined gout were obtained from Ref.1 (Nakayama, et al., ***Ann Rheum Dis***, 2020)

^e^ Risk allele is defined as a base which increases SU level and gout risk

^f^ Log_10_ (Bayes’ factor) of > 6 was adopted for a genome-wide significance level (Ref.2)

^g^ The significance level α was set to a *p* value of < 1.19 × 10^–3^ (= 0.05/42 with Bonferroni correction)

Loci identified for the first time in clinically-defined gout cases are shown in **bold**. Of three loci, two loci including *ORC4* and *MYO9A* were identified as novel gout loci

*SNP* Single nucleotide polymorphism, *Chr* Chromosome, *SU* Serum urate, *BF* Bayes’ factor*, OR* Odds ratio, *95%CI* 95% Confidence interval

We identified rs2307394 of *ORC4* as a novel gout locus (2q23.1)*.* ORC4 is one of the origin recognition complexes that binds specific DNA replication origins to initiate DNA synthesis [6]. However, its physiological and pathophysiological functions on urate handling and/or susceptibility to gout remain unclear. Another gene at the same locus (2q23.1), *ACVR2A*, encodes a receptor protein involved in the signaling of activins, which form part of the transforming growth factor-beta (TGF-β) family. TGF-β signaling mediates a urate-induced pro-inflammatory phenotype in human monocytes [7], suggesting that *ACVR2A* might play a role in urate handling and gout development. The present study suggests, for the first time, an association between gout and *ACVR2A*.

**Fig. 1:**
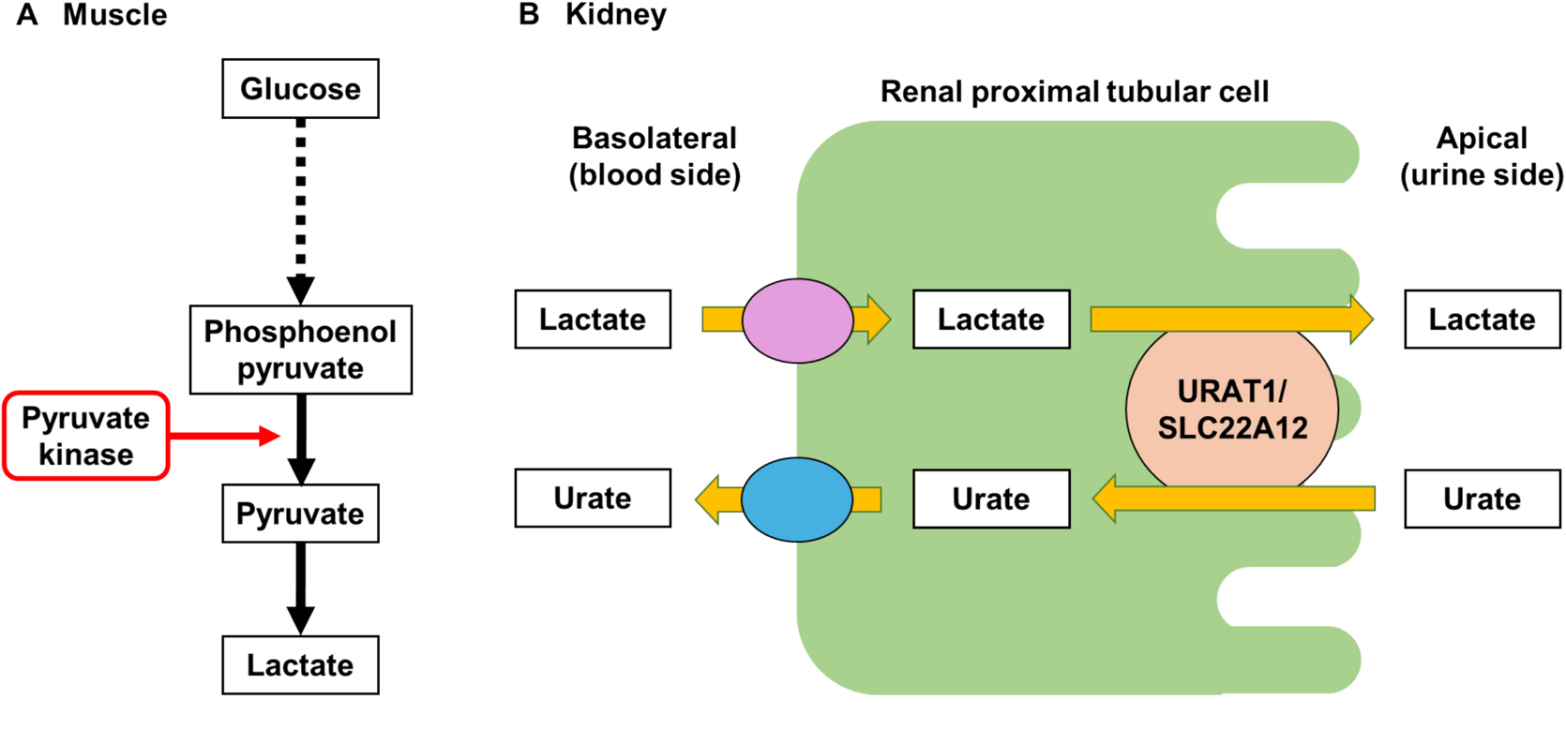
Pyruvate kinase encoded by *PKM* relates to renal urate reabsorption In the glycolytic system in the tissues such as muscle (A), pyruvate kinase ultimately metabolizes phosphoenolpyruvate to pyruvate, which results in an increase in lactate. In the kidney (B), lactate is known to enhance renal urate reabsorption via urate transporter 1 (URAT1/SLC22A12), which leads to an increase in serum urate levels and gout susceptibility

We also identified rs2957742 of *MYO9A* as a novel gout locus (15q23)*. MYO9A* encodes an atypical myosin that functions as an actin-based molecular motor. It was recently identified as a shared gene signature between rheumatoid arthritis (RA) and colorectal cancer [8]. Given that gout and RA share similar mechanisms such as IL-1β and inflammasome activation, *MYO9A* might also be associated with gout through a similar inflammatory pathway to that of RA. Another gene at the same locus (15q23), *pyruvate kinase M1/M2* (*PKM,* also known as “*pyruvate kinase, muscle*”), encodes pyruvate kinase M1/M2, a key enzyme in glycolysis [9]. This enzyme catalyzes the transfer of a phosphoryl group from phosphoenolpyruvate to ADP, producing ATP and pyruvate (Fig. 1A), which results in an increase in lactate. Lactate enhances renal urate reabsorption activity via urate transporter 1 (URAT1), which should lead to an increase in serum urate levels and gout susceptibility (Fig. 1B). This is the first report to suggest an association between gout and *PKM.*

We for the first time revealed rs4886755 of *NRG4* to be a locus (15q24.2) that is significantly associated with clinically defined gout. NRG4 is an adipokine that is primarily secreted by brown adipose tissue. It plays a significant role in regulating energy homeostasis and glucolipid metabolism, and protects against the development of non-alcoholic fatty liver disease (NAFLD) [10]. Oxidative stress, which causes inflammation and hepatic lipotoxicity, is a leading cause of NAFLD. In a previous report, we suggested *PNPLA3* to be associated with serum urate levels, since *PNPLA3*, which is also associated with NAFLD, is related to inflammation and oxidative stress [2]. *NRG4* is, therefore, also likely to be associated with serum urate levels and to have protective effects against the inflammation and oxidative stress that causes NAFLD; it thus may have a significant association with gout. The present study is the first to posit an association between gout and *NRG4*.

In conclusion, the present study indicates the significant association of *ORC4*, *MYO9A*, and *NRG4* with clinically-defined gout, with *ORC4* and *MYO9A* being identified as novel gout loci. We also suggest that other genes at these three loci—*ACVR2A* and *PKM—*could be involved in the pathogenesis of gout. While further studies will be needed on these loci, our approach and findings should lead to a better understanding of the molecular pathogenesis that underlies the development of gout.

## Supplementary Information

Below is the link to the electronic supplementary material.

Supplementary file1 (XLSX 22 KB)

## Data Availability

Data are available upon reasonable request to the corresponding author.
